# Gemeinschaftsdiagnose: Pandemie verzögert Aufschwung — Demografie bremst Wachstum

**DOI:** 10.1007/s10273-021-2915-4

**Published:** 2021-05-17

**Authors:** Oliver Holtemöller, Stefan Kooths, Claus Michelsen, Torsten Schmidt, Timo Wollmershäuser

**Affiliations:** 1 Abteilung Makroökonomik, Institut für Wirtschaftsforschung Halle, Kleine Märkerstraße 8, 06108 Halle (Saale), Deutschland; 2 Leiter Prognosezentrum, Institut für Weltwirtschaft, Kiellinie 66, 24105 Kiel, Deutschland; 3 Konjunkturpolitik, DIW Berlin, Mohrenstr. 58, 10117 Berlin, Deutschland; 4 RWI - Leibniz-Institut für Wirtschaftsforschung, Hohenzollernstraße 1-3, 45128 Essen, Deutschland; 5 und Befragungen, ifo Zentrum für Konjunkturforschung, Poschingerstraße 5, 81679 München, Deutschland

**Keywords:** E32, E66, J11

## Abstract

Das erste Jahr der Corona-Pandemie stand in Deutschland im Zeichen extremer Schwankungen der ökonomischen Aktivität und einer massiv gelähmten Binnenwirtschaft. In ihrem Frühjahrsgutachten gehen die Institute davon aus, dass der derzeitige Shutdown zunächst fortgesetzt und ab Mitte Mai bis zum Ende des dritten Quartals nach und nach aufgehoben wird. Im Zuge der Lockerungen wird sich vor allem der private Konsum kräftig erholen. Insgesamt dürfte das Bruttoinlandsprodukt in diesem Jahr um 3,7 % und im kommenden Jahr um 3,9 % zulegen.

## Abstract (en)

In Germany, the first year of the coronavirus pandemic was characterised by extreme fluctuations in economic activity and a massively paralysed domestic economy. In their spring report, the leading economic research institutes assume that the current shutdown will continue and gradually be lifted from mid-May until the end of the third quarter. In the wake of the easing, private consumption in particular will recover strongly. Overall, GDP is expected to grow by 3.7 % this year and 3.9 % next year.

Das erste Jahr der Corona-Pandemie stand im Zeichen extremer Schwankungen der ökonomischen Aktivität und einer massiv gelähmten Binnenwirtschaft. Anders als in früheren Krisen ist der private Konsum diesmal kein stabilisierender Faktor, sondern seinerseits mitursächlich für die starken Schwankungen. Maßgeblich hierfür ist, dass Infektionsschutzmaßnahmen zahlreiche kontaktintensive Geschäftsmodelle vor allem in den konsumbezogenen Dienstleistungsbranchen behindern, sodass die privaten Haushalte ihre Ausgaben nicht wie gewohnt tätigen können. Im Zuge der zweiten Infektionswelle während des zurückliegenden Winterhalbjahrs gab die Aktivität in den konsumnahen Dienstleistungsbereichen erneut deutlich nach. Dagegen war die Industriekonjunktur vor allem dank eines steigenden Auslandsgeschäfts bis zuletzt weiter aufwärts gerichtet.

Trotz des immer wieder verlängerten zweiten Shutdowns haben sich eine Reihe von Indikatoren zuletzt wieder deutlich verbessert. Dabei ist bemerkenswert, dass sich die Geschäftserwartungen des ifo Instituts auch im Dienstleistungssektor und im Handel aufgehellt haben. Darin dürfte vor allem die Erwartung zum Ausdruck kommen, dass die zunehmende Immunisierung der Bevölkerung die Infektionsschutzmaßnahmen in absehbarer Zeit entbehrlich macht. Allerdings gehen die Institute davon aus, dass der derzeitige Shutdown zunächst fortgesetzt wird und dabei auch die zuletzt erfolgten Lockerungen wieder weitgehend zurückgenommen werden. Erst ab Mitte des zweiten Quartals setzen Lockerungsschritte ein, die es den im Shutdown befindlichen Unternehmen erlauben, ihre Aktivitäten nach und nach wieder aufzunehmen. Bis zum Ende des dritten Quartals sollten dann alle Beschränkungen aufgehoben worden sein, weil bis dahin insbesondere mit einem weitreichenden Impffortschritt zu rechnen ist.

**Prof. Dr. Oliver Holtemöller** ist stellvertretender Präsident und leitet die Abteilung Makroökonomik am Leibniz-Institut für Wirtschaftsforschung Halle.

**Prof. Dr. Stefan Kooths** leitet das Prognosezentrum am Institut für Weltwirtschaft in Kiel.

**Dr. Claus Michelsen** leitet die Abteilung Konjunkturpolitik am Deutschen Institut für Wirtschaftsforschung in Berlin (DIW Berlin).

**Prof. Dr. Torsten Schmidt** ist Leiter des Kompetenzbereichs „Wachstum, Konjunktur, Öffentliche Finanzen" am RWI – Leibniz-Institut für Wirtschaftsforschung in Essen.

**Prof. Dr. Timo Wollmershäuser** ist stellvertretender Leiter des ifo Zentrums für Makroökonomik und Befragungen in München.

Unter dieser Voraussetzung wird es in den Dienstleistungsbereichen zu einer kräftigen Ausweitung der wirtschaftlichen Aktivität im Sommerhalbjahr kommen. Verwendungsseitig spielt dabei der private Konsum die Hauptrolle. Die Erfahrungen aus dem vergangenen Frühjahr legen nahe, dass sich das wirtschaftliche Geschehen in vielen Bereichen rasch normalisiert und der Aufholprozess im Verlauf dieses Jahres weitgehend abgeschlossen sein wird. Im kommenden Jahr fallen die Zuwachsraten des Bruttoinlandsprodukts dann wieder normal aus.

Infolge der für das Sommerhalbjahr erwarteten kräftigen Erholung dürfte das Bruttoinlandsprodukt in diesem Jahr um 3,7 % zulegen. Die mit 3,9 % sogar noch etwas höhere Rate im kommenden Jahr geht überwiegend auf die kräftigen Zuwächse in der zweiten Jahreshälfte 2021 zurück (die Jahresverlaufsrate 2022 beträgt 1,4 %).

Die bisher niedrigen Zahlen bei den Unternehmensinsolvenzen dürften vor allem auf die staatlichen Unterstützungsmaßnahmen und das Aussetzen der Anmeldepflicht für Insolvenzen zurückzuführen sein, welches derzeit noch bis Ende April dieses Jahres vorgesehen ist. Durch den Corona-Schock sind die Geschäftsmodelle der davon betroffenen Wirtschaftsbereiche für die Zeit nach der Pandemie ganz überwiegend nicht obsolet geworden, sondern dürften dann weiterhin marktfähig sein. Allerdings steigt die Zahl der Insolvenzanmeldungen auch in normalen Rezessionen erst mit einer gewissen zeitlichen Verzögerung. Weil ein Teil der Anzeigen diesmal durch die wirtschaftspolitischen Maßnahmen verhindert wurde, wird es wohl vorübergehend zu einem deutlichen Anstieg der Insolvenzen kommen, wenn die staatlichen Hilfsmaßnahmen auslaufen und die Pflicht zur Insolvenzanmeldung wieder vollumfänglich gilt.

## Mittelfristige Wirtschaftsentwicklung zunehmend im Zeichen des demografischen Wandels

Die Folgen der Corona-Krise dürften die Wirtschaftsaktivitäten auch mittelfristig belasten. Das aktuell geschätzte Produktionspotenzial liegt in den Jahren 2020 bis 2024 durchschnittlich rund 1,1 % unter dem Niveau, das vor der Corona-Krise (Herbstgutachten 2019) geschätzt wurde. Ursächlich für diese Abwärtsrevision sind insbesondere Revisionen des potenziellen Arbeitsvolumens und des Trends der totalen Faktorproduktivität (TFP). In der kurzen Frist überwiegt dabei der Revisionsbeitrag des Arbeitsvolumens, in den Jahren danach der Revisionsbeitrag der TFP. Der von der Fortschreibung des Kapitalstocks ausgehende Revisionsbeitrag ist hingegen verhältnismäßig gering und am Ende der mittleren Frist aufgrund der veränderten Prognose für die Investitionstätigkeit sogar leicht positiv.

Die Revision des Produktionspotenzials im Zuge der Corona-Krise fällt damit im Vergleich zu früheren schweren Wirtschaftskrisen gering aus.[1] So hat die Europäische Kommission das deutsche Produktionspotenzial für das Jahr 2010 zwischen November 2008 und Mai 2009 um 3 % herabgesetzt. Die vergleichsweise geringe Revision in diesem Gutachten nach einem ähnlich starken Einbruch der Wirtschaftsleistung unterstellt, dass die bestehenden Produktionsstrukturen nicht in beträchtlichem Umfang obsolet geworden sind. Allerdings sind Schätzungen des Produktionspotenzials mit großer Unsicherheit behaftet und die mittel- und langfristigen Folgen der Corona-Krise sind noch schwer absehbar.

Im Unterschied dazu ist inzwischen deutlich erkennbar, dass die Entwicklung des Produktionspotenzials in den kommenden Jahren stark von der demografischen Entwicklung geprägt wird. Die Einschätzung der zukünftigen Bevölkerungsentwicklung lehnt sich an die Variante 2 der 14. koordinierten Bevölkerungsvorausberechnung des Statistischen Bundesamts an (G2-L2-W2: moderate Entwicklung der Geburtenhäufigkeit, Lebenserwartung und Wanderung). Nach den Berechnungen der Institute ergibt sich daraus für das potenzielle Arbeitsvolumen bis zum Jahr 2025 ein jahresdurchschnittlicher Rückgang um 0,1 %. Nach Anstiegen in den Jahren 2020 und 2021 um jeweils 0,3 % setzt ab dem Jahr 2023 demografisch bedingt der Schrumpfungsprozess ein, wodurch zugleich das Wachstum des Produktionspotenzials sinkt.

Alles in allem steigt das Produktionspotenzial nach den Berechnungen der Institute bis zum Jahr 2024 um durchschnittlich 0,9 % pro Jahr. Die Wachstumsrate liegt damit etwa 0,4 Prozentpunkte unter dem Mittel seit 1996.

## Folgen des demografischen Wandels für das Wirtschaftswachstum

Der sich bereits in der mittleren Frist abzeichnende demografische Wandel wird sich in den darauf folgenden Jahren weiter verstärken. Ausschlaggebend hierfür ist, dass ein Großteil der etwa 13 Mio. Babyboomer – der geburtenstarken Jahrgänge zwischen Mitte der 1950er Jahre und Mitte der 1960er Jahre – das Rentenalter erreicht. Somit steigt der Anteil der Rentner:innen bis 2030 deutlich an, während die Erwerbsbevölkerung schrumpft. Dies wirkt sich über zwei Kanäle auf das Wirtschaftswachstum aus. Zum einen sinkt das Arbeitsangebot mit der abnehmenden Zahl von Personen

1 Für eine Analyse der Revisionen der Potenzialschätzung in verschiedenen zyklischen Phasen und vor und nach Finanzkrisen vgl. Ademmer et al. (2019).

im erwerbsfähigen Alter. Zum anderen deuten Studien darauf hin, dass die Verschiebung der Altersstruktur die Arbeitsproduktivität negativ beeinflusst.

Um diesen Struktureffekt zu quantifizieren, verwenden die Institute ein vektorautoregressives (VAR) Modell mit zusätzlichen exogenen Variablen in Anlehnung an Aksoy et al. (2019). Die Ergebnisse zeigen, dass ein Anstieg des Anteils der Erwerbsbevölkerung (15 bis 39 Jahre und 40 bis 64 Jahre) langfristig einen positiven Effekt auf Wirtschaftswachstum, Investitionen, Ersparnis und Arbeitsstunden hat, während ein Anstieg des Anteils älterer Menschen (65 Jahre und älter) sich negativ auf diese Variablen auswirkt. Ein um einen Prozentpunkt höherer Anteil der Älteren lässt das Wachstum des Bruttoinlandsprodukts langfristig um 0,15 Prozentpunkte sinken, während ein um einen Prozentpunkt geringerer Anteil der 40- bis 65-Jährigen die jährliche Wachstumsrate um 0,03 Prozentpunkte dämpft. In der Summe würde eine solche Verschiebung der Altersstruktur das Wachstum um 0,18 Prozentpunkte verringern. Für die makroökonomischen Effekte des demografischen Wandels ist somit nicht nur das geringere Erwerbspersonenpotenzial, sondern auch die nach Alterskohorten veränderte Bevölkerungszusammensetzung entscheidend.

Abbildung 1

**Projektion der Veränderung der Wachstumsrate des Bruttoinlandsprodukts**

in Prozentpunkten

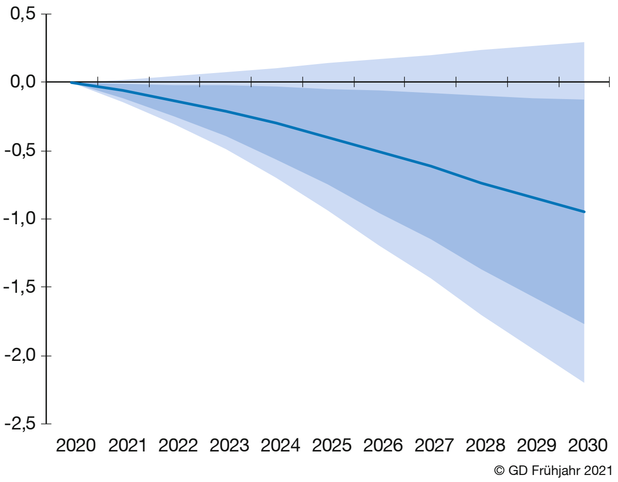

Anmerkungen: Einseitiges 80 %-Konfidenzintervall (dunkleres blau). Einseitiges 90 %-Konfidenzintervall (hellblau).

Quelle: Berechnungen der Institute.

Bis zum Jahr 2030 ergeben sich in Deutschland erhebliche Verschiebungen in der Altersstruktur.[2] Demzufolge fällt ausweislich der geschätzten Kompositionseffekte das Wachstum des Bruttoinlandsprodukts im Jahr 2030 um rund einen Prozentpunkt niedriger aus als im Jahr 2020 (vgl. Abbildung 1). Im Vergleich dazu blieb das Wachstum des Bruttoinlandsprodukts in den Jahren von 2010 bis 2020 nach dieser Schätzung infolge des demografischen Wandels nahezu unberührt.

Ursächlich für den langfristigen Rückgang der Wachstumsrate des Bruttoinlandsprodukts in der Projektion bis zum Jahr 2030 ist vor allem die Verschiebung der Altersstruktur. Das Wachstum der Gesamtbevölkerung sinkt dagegen nur geringfügig, wodurch das Wachstumspotenzial über den Altersstruktureffekt hinaus nur um etwa 0,1 Prozentpunkte gedämpft wird.

Im Rahmen des Modells kann auch der Frage nachgegangen werden, wie den dämpfenden Effekten des demografischen Wandels auf das Wirtschaftswachstum begegnet werden kann. Da die Demografie größtenteils durch die Geburtenraten der Vergangenheit determiniert ist, erscheinen insbesondere ein Anstieg der Migration oder ein späterer Renteneintritt als Möglichkeiten, dem Sinken der Erwerbsbevölkerung entgegenzuwirken. Legt man für die Bevölkerungsvorausberechnung einen um 100.000 Personen höheren Wanderungssaldo von dann 300.000 Nettozuwanderern zugrunde (Variante G2-L2-W3), so schwächt sich der Anstieg des Anteils der über 64-Jährigen von 4,0 auf 3,7 Prozentpunkte ab. Gleichzeitig sinkt der Anteil der 15- bis 64-Jährigen weniger stark und die Gesamtbevölkerung steigt leicht. Zusammengenommen könnte eine etwas höhere Migration damit den Rückgang des Potenzialwachstums um gut 0,1 Prozentpunkt mildern.

Für die Simulation eines erhöhten Renteneintrittsalters unterstellen wir, dass der Produktionsbeitrag der Älteren genauso groß ist wie jener der 40- bis 64-Jährigen. Zur Quantifizierung der Effekte einer Anhebung des Renteneintrittsalters auf 70 Jahre wird der Anteil der 40- bis 64-Jährigen um den Anteil der 65- bis 69-Jährigen erhöht und der Anteil dieser Kohorte auf null gesetzt. In gleicher Weise kann eine Anhebung des Renteneintrittsalters auch auf 75 Jahre simuliert werden, indem der Anteil der 65- bis 74-Jährigen der Kohorte der 40- bis 64-Jährigen zugeschlagen wird. Insbesondere bei diesem Szenario sind die Annahmen bezüglich der Partizipationsquote und Arbeitsproduktivität optimistisch, sodass die Ergebnisse als Obergrenze des möglichen

2 Den Berechnungen liegt die mittlere Variante der 14. Koordinierten Bevölkerungsvorausberechnung zugrunde. Darin bleibt der Anteil der 0- bis 14-Jährigen nahezu konstant (+0,5 Prozentpunkte), jedoch fällt der Anteil der 15- bis 64-Jährigen deutlich (-4,5 Prozentpunkte), während jener der über 64-Jährigen stark steigt (+4,0 Prozentpunkte).

Potenzialeffektes zu interpretieren sind. Bei einer Anhebung des Renteneintrittsalters auf 70 Jahre würde dementsprechend das Potenzialwachstums um 0,3 Prozentpunkte und bei einer Anhebung auf 75 Jahre um 0,5 Prozentpunkte weniger stark zurückgehen als im Basisszenario (vgl. Abbildung 2).

Abbildung 2
**Auswirkungen der Verschiebung des Renteneintrittsalters auf Potenzialwachstum**
in Prozentpunkten

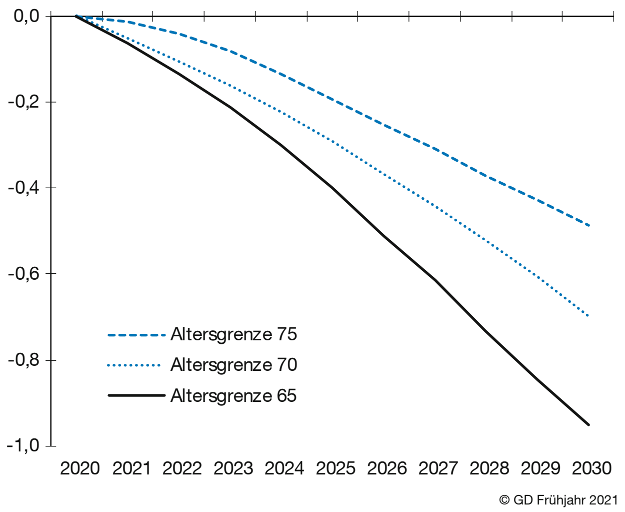

Quelle: Berechnungen der Institute.

## Schlussfolgerungen

Auch wenn die Corona-Pandemie in Deutschland in den kommenden Monaten nach und nach eingedämmt werden dürfte, werden die Folgen noch geraume Zeit spürbar sein. Die Wirtschaftspolitik hat zügig und mit umfangreichen Maßnahmen auf die wirtschaftliche Krise reagiert. Dies dürfte im Großen und Ganzen dazu beigetragen haben, dass der Bestand vieler durch die Krise getroffenen Unternehmen bislang gesichert wurde. Dabei spricht die Anatomie der Krise für solche Instrumente, die schnell und zielgerichtet wirken und sich zudem selbst dosieren. Hierzu zählt – neben dem Kurzarbeitergeld – die Möglichkeit des steuerlichen Verlustrücktrags, der bereits jetzt genutzt wird, allerdings auf die Gewinneinkünfte des Jahres 2019 begrenzt und bis zu einem Höchstbetrag von 10 Mio. Euro gedeckelt ist. Eine Ausweitung dieser Regelungen auf die Jahre 2018 und 2017 könnte die Liquidität der durch die Krise getroffenen Unternehmen ohne weitere Prüfung stärken. Mit den verschiedenen Corona-Hilfsprogrammen (Soforthilfe, Überbrückungshilfe, November-/Dezemberhilfe) kamen den Unternehmen auch Zuschüsse zugute, die jedoch nicht nur mehrfach nachjustiert wurden, sondern auch bürokratisch schwerfällig waren. Zudem lassen die Kriterien (Pauschalzahlungen, Fixkostenkompensation, Umsatzersatz) keine zielgenaue Stabilisierungswirkung erwarten (Felbermayr und Kooths, 2020).

Für junge und neu gegründete Unternehmen bedarf es darüber hinaus gesonderter Programme. Die Bundesregierung hat einen Fonds für Wagniskapital aufgelegt, der Start-ups und Scale-ups unterstützen soll. Allerdings machen die hierfür infrage kommenden Unternehmen nur einen kleinen Teil des Gründungsgeschehens in Deutschland aus. Um Unternehmensgründungen zu fördern, wäre beispielsweise auch eine Weiterentwicklung des Gründungszuschusses eine Handlungsmöglichkeit (Kritikos, 2011; Caliendo, 2016; Belitz et al., 2020). Bürokratische Hürden, welche die Dauer und die Kosten von Gründungen erhöhen, sollten daher deutlich gesenkt werden. Die zunehmende Verschuldung und schwächere Eigenkapitalposition drücken für sich genommen die Investitionstätigkeit der Unternehmen. Ein bewährtes Instrument zur Stärkung der unternehmerischen Investitionstätigkeit sind beschleunigte steuerliche Abschreibungsmöglichkeiten (Bofinger et al., 2020).

Darüber hinaus stellt der bevorstehende demografische Wandel die Wirtschaftspolitik vor Herausforderungen. Um dieser Entwicklung entgegenzuwirken, gibt es unterschiedliche Handlungsmöglichkeiten: So könnte Zuwanderung in jüngere Alterskohorten das Erwerbspersonenpotenzial erhöhen. Derzeit gehen die Projektionen im Mittel jedoch davon aus, dass pro Jahr lediglich 220.000 Personen insgesamt mehr ein- als auswandern (Statistisches Bundesamt, 2019). Daran dürfte auch das am 1.3.2020 verabschiedete Fachkräfteeinwanderungsgesetz, das die Erwerbszuwanderung aus Drittstaaten erleichtern soll, nicht viel ändern (Brückner et al., 2019). Die Verbesserung der Anerkennung im Ausland erworbener beruflicher Abschlüsse könnte dem entgegenwirken (Brückner et al., 2021).

Zudem könnte das Renteneintrittsalter nach 2029 weiter schrittweise angehoben werden. Angesichts einer steigenden Lebenserwartung und Vitalität wäre es sinnvoll, die zusätzliche Lebenszeit nicht nur als Ruhestandszeit, sondern anteilig für zusätzliche Arbeitszeit zu verwenden. Der erheblichen Heterogenität in der individuellen Lebenserwartung müsste dabei in geeigneter Form Rechnung getragen werden. Eine Erhöhung des Renteneintrittsalters auf 69 Jahre würde die absehbare Reduktion des Potenzialwachstums um mehrere Zehntel Prozentpunkte in den 2020er Jahren mildern und den absehbaren drastischen Anstieg der Bundeszuschüsse zur Rentenversicherung reduzieren. Ein Verzicht darauf würde den finanziellen Handlungsspielraum des Bundes in absehbarer Zeit erheblich einengen.
